# RACK1 promotes miR-302b/c/d-3p expression and inhibits CCNO expression to induce cell apoptosis in cervical squamous cell carcinoma

**DOI:** 10.1186/s12935-020-01435-0

**Published:** 2020-08-10

**Authors:** Jing Wang, Shengcai Chen

**Affiliations:** Department of Gynaecology, Affiliated Hospital of Youjiang Medical University for Nationalities, No. 18, Zhongshan Second Road, Youjiang District, Baise, Guangxi Zhuang Autonomous Region 533000 People’s Republic of China

**Keywords:** Cervical squamous cell carcinoma, Receptor for activated protein C kinase 1, Cyclin O, MicroRNA-302b/c/d-3p, Apoptosis

## Abstract

**Background:**

Cervical squamous cell carcinoma (CSCC) is one of the main causes of cancer-related deaths in women worldwide. The present study was conducted with the main objective of determining the potential role of receptor for activated protein kinase C1 (RACK1) in CSCC through regulation of microRNA (miR)-302b/c/d-3p and Cyclin O (CCNO).

**Methods:**

The expression of RACK1, miR-302b/c/d-3p and CCNO in CSCC tissues and cells was measured by RT-qPCR and Western blot analysis. The interaction among RACK1, miR-302b/c/d-3p, and CCNO was determined by dual luciferase reporter assay. Subsequently, effects of RACK1, miR-302b/c/d-3p and CCNO on CSCC cell cycle entry, proliferation and apoptosis were investigated with the use of flow cytometry, EdU, and TUNEL assays. Furthermore, mouse xenograft model of CSCC cells was established to verify the function of RACK1 in vivo.

**Results:**

RACK1 and miR-302b/c/d-3p were down-regulated and CCNO was overexpressed in CSCC. CCNO was identified as the target of miR-302b/c/d-3p. Importantly, overexpressed miR-302b-3p, miR-302c-3p or miR-302d-3p or RACK1 enhanced the apoptosis and suppressed the proliferation of CSCC cells in vitro, while inhibiting tumor growth in vivo by targeting CCNO.

**Conclusions:**

On all accounts, overexpressed RACK1 could dampen the progression of CSCC through miR-302b/c/d-3p-mediated CCNO inhibition.

## Background

Cervical squamous cell carcinoma (CSCC) is one of the causes of cancer-related deaths in women [1]. There were 569,847 newly diagnosed cases of CSCC and 311,365 deaths reported in 2018 [2]. Notably, CSCC has been proven to be the second leading cause of cancer deaths in young women (aged 20 to 39 years), which highlights that increased screening and administration of human papillomavirus (HPV) vaccination are of a high necessity in young women [3]. The therapeutic ratio of CSCC can be significantly improved via image-guided brachytherapy, as it reduces late toxicities [4]. A low pretreatment HPV viral load may indicate poor prognosis in CSCC, and the survival nomogram based on it can estimate the long-term prognosis [5].

Transcription factor AP-1 is involved in cervical cancer development [6]. Another transcription factor, homeobox D9 is also associated with malignant phenotype of cervical cancer [7]. Receptor for activated protein kinase C (RACK1), a multifunctional scaffolding protein, plays a functional role in nucleating cell signaling hubs and regulating protein activity, and is also involved the modulation of migration and invasion of tumor cells [8]. Moreover, downregulated RACK1 leads to pancreatic cancer growth and metastasis [9]. RACK1 depletions can also induce metastasis of gastric cancer (GC) by promoting the microRNA (miR)-302c/interleukin (IL)-8 axis [10]. microRNAs (miRNAs) are endogenous non-coding RNAs, and multiple tumors have been observed to have dysregulated levels of miRNAs [11]. For instance, it is illustrated that miR-373 can promote the apoptosis of CSCC SiHa cells [12]. miR-195 has also been found to suppress proliferation of human cervical cancer cells by targeting cyclin D1 [13]. The miR-302-b/c/d has been found to exert crucial effects on a variety of biological processes, and modulates multiple pathological changes including cancer [14]. miR-302-3p has been shown to inhibit cervical cancer cell migration and invasion by directly targeting defective in cullin neddylation 1 domain containing 1 (DCUN1D1) [15]. In the current study, miR-302b/c/d-3p was found to bind to the 3′-untranslated region (3′-UTR) of Cyclin O (CCNO) mRNA and then directly targeted it. CCNO is a small gene encoding a 1053 bp cDNA and a 350-amino-acid protein, and comprises two cyclin box folds [16]. CCNO has been identified as a novel protein of the cyclin family, and is known to be involved in the regulation of oocyte meiotic progression at different stages [17]. Its down-regulation inhibits the tumorigenicity of GC by enhancing cell apoptosis [18]. These findings led to the hypothesis that RACK1 might participate in the development of CSCC through the regulation of miR-302b/c/d-3p and CCNO.

## Materials and methods

### Ethics statement

This study was approved by the Ethic Committee of Affiliated Hospital of Youjiang Medical University for Nationalities and performed in strict accordance with the *Declaration of Helsinki*. All participants signed informed consent documentation prior to the study. Animal experiments were approved by the Institutional Animal Care and Use Committee of Affiliated Hospital of Youjiang Medical University for Nationalities with extensive efforts made to minimize animal suffering during the study.

### Study subjects

Clinical CSCC samples were collected from 46 patients with CSCC who underwent hysterectomy from August 2015 to July 2016. The inclusion criteria for selection of patients were as follows: the patients did not receive chemotherapy, radiotherapy, endocrine therapy or other anti-tumor treatment before surgery; the patients were pathologically confirmed as CSCC after surgery; the patients had complete medical records and follow-up data. Patients suffering from combined diseases including combined breast cancer, ovarian cancer, severe liver and kidney dysfunction were excluded from the study. Thirty cases of normal cervical tissues were collected from patients who underwent hysterectomy for benign gynecological diseases.

### Cell treatment

The CSCC cell line CasKi and the human normal cervical epithelial immortalized cell line H8 were purchased from the cell bank of BeNa Culture Collection (BNCC). Cells were cultured in Eagle’s minimal essential medium (EMEM) supplemented with 10% fetal bovine serum (FBS) and 1% penicillin/streptomycin at 37 °C with 5% CO_2_ and 95% saturated humidity. Cells at passage 3 were inoculated into a 24-well plate at a density of 2 × 10^6^ cells/well), and cultured to grow into monolayer cells. CasKi cells were treated with following plasmids: small interfering RNA-negative control (si-NC), si-CCNO, NC-mimic, miR-302b-3p mimic, miR-302c-3p mimic, miR-302d-3p mimic, overexpression (oe)-NC, or oe-RACK1 using Lipofectamine 2,000 (Invitrogen Inc., Carlsbad, CA, USA). All plasmids were constructed by Shanghai Sangon Biotech company (Shanghai, China).

### Immunohistochemistry

The prepared paraffin sections were dewaxed and hydrated. After microwave antigen retrieval using 1 mM ethylenediaminetetraacetic acid (EDTA) (pH 8.0), the sections were added with 3% H_2_O_2_-methanol. Next, the sections were added with primary antibody against CCNO (ab47682, 1:500, Rabbit, Abcam, Cambridge, UK), and RACK1 (5432, 1:1000, Rabbit, Cell Signaling Technologies, Beverly, MA, USA), and incubation was carried out overnight at 4 °C. Thereafter, the sections were re-probed with polymer enhancer (PV-9000, ZSGB-Bio, Beijing, China) at room temperature for 20 min. Next, the sections underwent further incubation with enzyme-labeled anti-mouse/rabbit polymer (PV-9000, ZSGB-Bio, Beijing, China) at room temperature for 30 min, and developed using 3,3′-diaminobenzidine (DAB) for 5 min. After the development was halted by distilled water, the sections were counterstained with hematoxylin, differentiated and blued. The sections were conventionally hydrated, cleared and sealed. Finally, the sections were photographed and observed under an inverted microscope (CX41, Olympus, Tokyo, Japan).

### Reverse transcription quantitative polymerase chain reaction (RT-qPCR)

Total RNA was extracted from tissues or cells, followed by reverse transcription into complementary DNA (cDNA). Primers (Table 1) were designed and synthesized by Invitrogen (Invitrogen Inc., Carlsbad, CA, USA). With glyceraldehyde-3-phosphate dehydrogenase (GAPDH) and U6 (Invitrogen Inc., Carlsbad, CA, USA) used as internal references, RT-qPCR was carried out on an ABI 7,500 quantitative PCR instrument (Thermo Fisher Scientific Inc., Waltham, MA, USA) using the SYBR^®^ Premix Ex Taq™ (Tli RNaseH Plus) kit (RR820A, Takara Holdings Inc., Kyoto, Japan). The final data were analyzed using the 2^−ΔΔCt^ method.

**Table 1:** Primer sequences for RT-qPCR

Genes	Primer sequences (5′–3′)
RACK1	F: TCTCTTTCCAGCGTGGCCATTAGA
	R: CCTCGAAGCTGTAGAGATTCCGACAT
miR-302b-3p	F: ATCCAGTGCGTGTCGTG
	R: TGCTTAAGTGCTTCCATGTT
miR-302c-3p	F: GCGTGCTTCCATGTTTCAGTGG
	R: CAGTGCAGGGTCCGAGGTAT
miR-302d-3p	F: TCTACTTTAACATGGAGGCACTT
	R: TCACCAAAACATGGAAGCAC
CCNO	F: TCTACAGACCTTCCGCGACT
	R: GCTCTACCAGCACCTCACTT
U6	F: CGCTTCGGCAGCACATATACTA
	R: CGCTTCACGAATTTGCGTGTCA
GAPDH	F: TCATCTCTGCCCCCTCTGCTG
	R: GCCTGCTCACCACCTTCTTG

F, forward; R, reverse; RT-qPCR, reverse transcription quantitative polymerase chain reaction; RACK1, receptor for activated protein kinase C; CCNO, Cyclin O; GAPDH, glyceraldehyde-3-phosphate dehydrogenase

### Western blot analysis

Total protein was isolated from tissues or cells using radioimmunoprecipitation assay (R0010, Beijing Solarbio Science & Technology Co., Ltd. (Beijing, China). Then the protein was separated using 10% sodium dodecyl sulfate polyacrylamide gel electrophoresis and transferred to polyvinylidene fluoride membrane. Next, the membrane was blocked with Tris-buffered saline Tween-20 (TBST) solution containing 5% bovine serum albumin (BSA), and incubated at 4 °C overnight with the diluted primary anti-rabbit antibody as follows: GAPDH (5174, 1:1000, 36 KD), RACK1 (5432, 1:1000, 32 KD), CCNO (ab47682, 1:1000, 40 KD), B-cell lymphoma 2 (Bcl-2) (3498, 1:1000, Rabbit, 26 KD), Cyclin D1 (2922, 1:1000, 15 KD), cleaved poly (ADP-ribose) polymerase (PARP) (#5625, 1:1000, 89 KD), and cleaved caspase 3 (#9664, 1:1000, 17 KD) [19]. All of the aforementioned antibodies were purchased from Cell Signaling Technology (Beverly, MA, USA), with the exception of CCNO from Abcam, Cambridge, UK. Then, the membrane was incubated with secondary antibody goat anti-rabbit immunoglobulin G (IgG) (ab150077, 1:1000, Abcam, Cambridge, UK). Lastly, the membrane was developed using enhanced chemiluminescence and analyzed using the gel image analysis software Image J.

### Dual luciferase reporter assay

The synthetic 3′-UTR of CCNO wild type (WT) gene fragment was introduced into pMIR-reporter (Huayueyang Biotechnology Co., Ltd., Beijing China) using the endonucleases SpeI and HindIII, after which the mutant type (MUT) was designed based on the complementary sequence of 3′-UTR of CCNO WT. The target fragment was inserted into the pMIR-reporter reporter plasmid using T4 DNA ligase following restriction endonuclease digestion. The correctly sequenced luciferase reporter plasmids WT or MUT were co-transfected with miR-302b/c/d-3p mimic or NC mimic into HEK293T cells. The cells were then collected and lysed after 48 h of transfection, after which luciferase activity was detected with the use of Glomax 20/20 luminometer fluorescence detector (Promega, Madison, WI, USA) using a luciferase assay kit (K801-200, BioVision Technologies, Exton, PA).

### 5-Ethynyl-2′-deoxyuridine (EdU) assay

Cells were seeded into 96-well plates at a density of 5 × 10^3^ cells/well. After 6 h, the cells were incubated with 100 µL of EdU solution for 2 h, fixed, and incubated with 2 mg/mL glycine for 5 min. Next, the cells were incubated with 100 µL penetrant for 10 min, added with 100 µL 1× Apollo staining reaction solution for 30 min of incubation, washed with 100 µL penetrant and washed using 100 µL methanol for 5 min. Subsequently, the wells were incubated with 100 µL 1× Hoechst 33,342 reaction solution under dark conditions for 30 min on a rotary shaker, after which the cells were blocked by the addition of 100 µL anti-fluorescence quenching tablets. Lastly, cells were photographed under a fluorescence microscope (Olympus FV1000, Olympus, Tokyo, Japan), and the number of cells was recorded. If the nucleus of the cell stained red, it was labeled positive and the exception to this finding were negative cells.

### Flow cytometry

Cells were centrifuged and cell pellet was resuspended in phosphate-buffered saline (PBS) to a concentration of about 1 × 10^5^ cells/mL. Subsequently, the cells were fixed at 4 °C for 1 h using 1 mL pre-cooled − 20 °C 75% ethanol, and centrifuged, with the ethanol removed. The cells were subsequently added with 100 µL RNase A avoiding exposure to light, subjected to water bath at 37 °C for 30 min, added with 400 µL propidium iodide (PI) and incubated at 4 °C for 30 min. Finally, cell cycle entry was detected at 488 nm.

### Terminal deoxynucleotidyl transferase-mediated dUTP-biotin nick end labeling (TUNEL) assay

Tissues or cell slides were fixed, incubated with PBS containing 0.1% Triton X-100 on ice bath for 2 min, permeabilized, and then incubated with TUNEL solution at 37 °C for 60 min avoiding exposure to light. Next, the cells were sealed with anti-fluorescence quenching liquid and observed under a fluorescence microscope at 450–500 nm and 515–565 nm.

### Tumor xenograft in nude mice

Thirty BALB/c female nude mice (aged 5 week, with weight of 18–21 g) purchased from Shanghai Lingchang Company (Shanghai, China) were selected and bred in the specific pathogen free environment at the animal experiment center of Affiliated Hospital of Youjiang Medical University for Nationalities before experiment. The nude mice were bred for 7 days in comfortable environment with moderate temperature, fed with aseptic food and drinking water, and an alternation of 12 h day and night. The lentiviral vectors of NC agomir, miR-302b-3p agomir, miR-302c-3p agomir, miR-302d-3p agomir, oe-NC and oe-RACK1 were purchased from the Shanghai Sangon Biotech company (Shanghai, China). The lentivirus was collected and added to the CasKi cells (1 × 10^8^ TU/mL). Then the stably transfected CasKi cells were prepared as cell suspension (5 × 10^6^/mL) and subcutaneously injected into the right leg of nude mice. Tumor formation was observed daily. Once the tumor was evident, the long and short diameters of the tumor were measured every 5 d. After 40 d of feeding, the nude mice were euthanized by the administration of anaesthesia. Then, the subcutaneous tumors were removed, photographed and weighed.

### Statistical analysis

SPSS 21.0 statistical software (IBM Corp. Armonk, NY, USA) was used for data processing. The measurement data were expressed as mean ± standard deviation. Independent sample *t*-test was used for data comparison between two groups. One-way analysis of variance (ANOVA) was used for data comparison among multiple groups, followed by Tukey’s post hoc test. Repeated measures ANOVA was used for data comparison at different time points, followed by Bonferroni’s post hoc test with corrections for multiple comparisons. Pearson’s correlation coefficient was used to analyze the correlation between miR-302b/c/d-3p expression and CCNO expression. *p* < 0.05 indicated a statistically significant value.

## Results

### CCNO is highly expressed in CSCC, and down-regulated CCNO suppresses proliferation but promotes apoptosis of CSCC cells

**Fig. 1:**
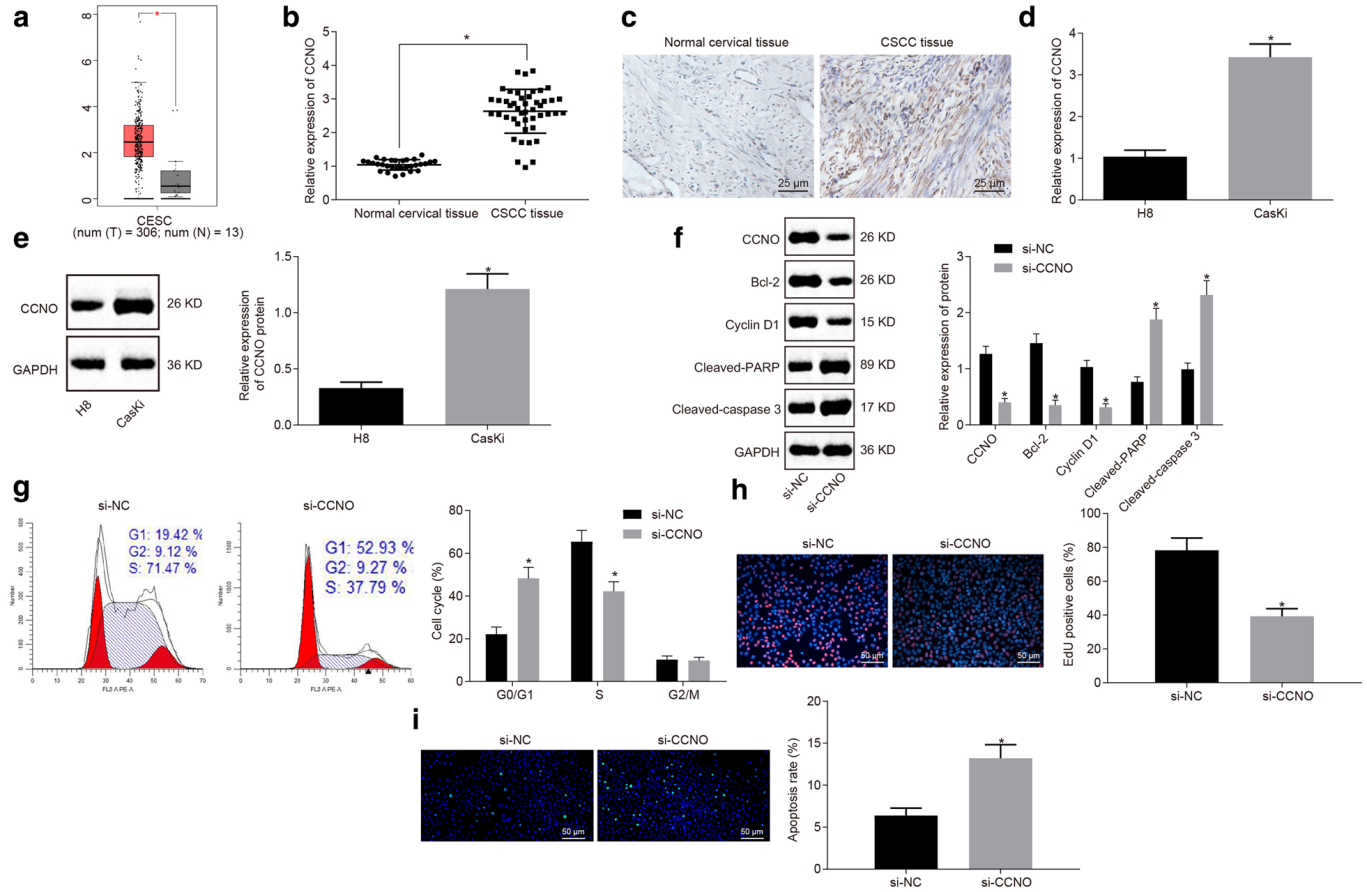
CCNO is up-regulated in CSCC, and down-regulated CCNO induces apoptosis of CSCC cells. **a** The expression of CCNO in CSCC and endocervical adenocarcinoma from GEPIA database; **b**, **c** CCNO expression in CSCC (N = 46) and normal cervical epithelial tissues (N = 30) detected by RT-qPCR and immunohistochemistry (× 400); **d **CCNO mRNA level in CSCC cell line (CasKi) and normal cell line (H8) detected by RT-qPCR; **e** The protein levels of CCNO, Bcl-2, Cyclin D1, cleaved PARP, cleaved caspase 3 in CSCC cell line (CasKi) and normal cell line (H8) detected by Western blot analysis, with the protein bands assessed; **f** The protein levels of CCNO, Bcl-2, Cyclin D1, cleaved PARP and cleaved caspase 3 in CasKi cells with CCNO silencing detected by Western blot analysis, with the protein bands assessed; **g** Cell cycle entry in CSCC cell line (CasKi) detected by flow cytometry; **h** Cell proliferation detected by EdU assay (×200); **i** Cell apoptosis detected by TUNEL assay (×200). *p < 0.05 vs. normal cervical epithelial tissues, H8 cell line or cells treated si-NC. The values in this figure were all measurement data and expressed as mean ± standard deviation. Independent sample *t* test was used for the data comparison between two groups. The experiment was repeated 3 times independently

The GEPIA database revealed a high expression of CCNO in CSCC and endocervical adenocarcinoma (*p* < 0.05) (Fig. 1a). RT-qPCR and immunohistochemistry were conducted to verify the upregulation of CCNO in CSCC, and the results showed an increase in CCNO expression in clinical samples of CSCC patients (both *p* < 0.05) (Fig. 1b, c. In addition, CCNO expression was evidently elevated in CSCC cell line (CasKi) (*p* < 0.05) (Fig. 1d, e).

Western blot analysis showed that downregulation of CCNO induced a marked decline in Bcl-2 and Cyclin D1 expression, while it resulted in elevated cleaved PARP and cleaved caspase 3 expression (*p* < 0.05) (Fig. 1f). Flow cytometry results showed that the number of cells arrested in the G0/G1 phase was increased and cells arrested in the S phase were obviously reduced in the absence of CCNO (*p* < 0.05) (Fig. 1g). EdU assay and TUNEL assay exhibited that downregulated CCNO led to decreased cell proliferation and increased cell apoptosis (both *p* < 0.05) (Fig. 1h, i). In summary, CCNO was highly expressed in CSCC, and down-regulated CCNO could promote apoptosis and inhibit proliferation of CSCC cells.

#### CCNO is a target gene of miR-302b/c/d-3p

**Fig. 2:**
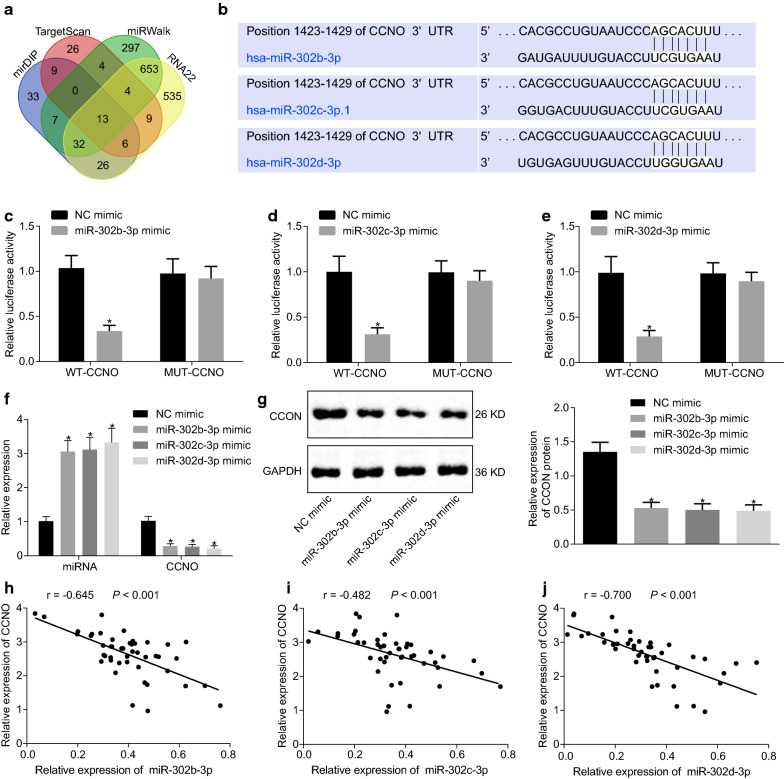
miR-302b/c/d-3p targets CCNO and inhibits its expression. **a** The comparison of upstream miRNA of CCNO predicted by RNA22, miRwalk, TargetScan, and mirDIP; **b **The binding site between miR-302b/c/d-3p and CCNO-3′-UTR; **c**–**e** Luciferase activity in cells after transfection with miR-302b/c/d-3p and WT-CCNO or miR-302b/c/d-3p and MUT-CCNO detected by dual luciferase reporter assay; **f** The expression of miR-302b/c/d-3p and CCNO in cells detected by RT-qPCR; **g** CCNO protein expression detected by Western blot analysis, with the protein bands assessed; **h**–**j** Correlation analysis between CCNO expression and miR-302b/c/d-3p expression in CasKi cells. *p < 0.05 vs. the cells treated with NC mimic. The values in the figure were measurement data, and expressed as mean ± standard deviation. Independent sample *t* test was used for data comparison between two groups. One-way ANOVA was used for data comparison among multiple groups, and followed by Tukey’s post hoc test. Pearson’s correlation coefficient was used for correlation analysis between indicators. The experiment was repeated 3 times independently

Upstream miRNAs of CCNO were predicted by RNA22, miRwalk, TargetScan, and mirDIP. Then, miR-302b-3p, miR-302c-3p and miR-302d-3p were verified to have a targeted regulatory relationship with CCNO (Fig. 2a). The targeted binding sites between miR-302b-3p/miR-302c-3p/miR-302d-3p and CCNO were predicted by the bioinformatics online site (Fig. 2b). Luciferase activity was decreased in cells co-transfected with miR-302b/c/d-3p and WT-CCNO (*p* < 0.05) (Fig. 2c–e). Besides, overexpressed miR-302b/c/d-3p resulted in the significant inhibition of CCNO expression in the CasKi cells (*p* < 0.05) (Fig. 2f, g). Correlation analysis showed that there exists a negative correlation between CCNO and miR-302b-3p, miR-302c-3p and miR-302d-3p expressions in CasKi cells (Fig. 2h–j). On the basis of the aforementioned, CCNO was a target gene of miR-302b-3p, miR-302c-3p and miR-302d-3p and could be negatively regulated by them.

#### miR-302b-3p, miR-302c-3p or miR-302d-3p suppresses proliferation and enhances apoptosis of CSCC cells by downregulating CCNO

**Fig. 3:**
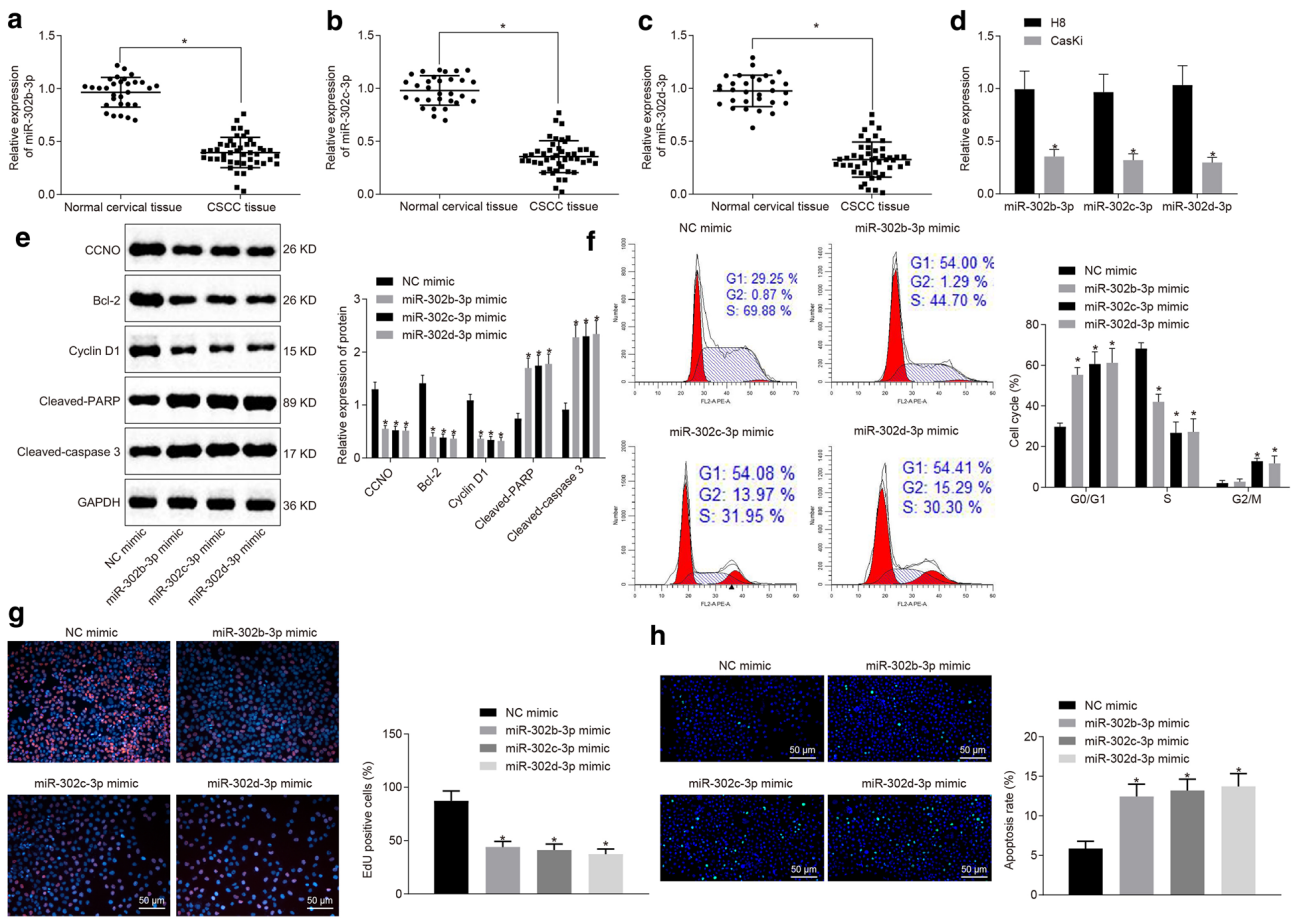
miR-302b-3p, miR-302c -3p or miR-302d-3p inhibits proliferation while inducing apoptosis of CSCC cells by targeting CCNO. **a**–**c** The expression of miR-302b/c/d-3p in CSCC (N = 46) and normal cervical tissues (N = 30) detected by RT-qPCR; **d** The expression of miR-302b/c/d-3p in CSCC cell line (CasKi) and normal cell line (H8) detected by RT-qPCR; **e** The protein levels of CCNO, Bcl-2, Cyclin D1, cleaved PARP, cleaved caspase 3 in CSCC cell line (CasKi) detected by Western blot analysis, with protein bands assessed; **f** The cell cycle entry in CSCC cell line (CasKi) detected by flow cytometry; **g** The cell proliferation detected by EdU assay (×200); **h** The cell apoptosis detected by TUNEL assay (×200). *p < 0.05 vs. normal cervical tissues, H8 cell line or cells treated with NC mimic. The values in the figure were all measurement data and expressed as mean ± standard deviation. Independent sample *t* test was used for data comparison between two groups, and one-way ANOVA with Tukey’s post hoc test was used for data comparison among multiple groups. The experiment was repeated 3 times independently

RT-qPCR showed a decrease in miR-302b-3p, miR-302c-3p and miR-302d-3p expressions in both CSCC patient tissues (*p* < 0.05) (Fig. 3a–c), and CasKi cell line (*p* < 0.05) (Fig. 3d). Then, miR-302b-3p, miR-302c-3p and miR-302d-3p were overexpressed in CSCC cell line. Western blot analysis showed that overexpressed miR-302b-3p, miR-302c-3p or miR-302d-3p led to reduced CCNO, Bcl-2 and Cyclin D1 expression and elevated cleaved PARP and cleaved caspase 3 expression (*p* < 0.05) (Fig. 3e). Moreover, overexpressed miR-302b-3p, miR-302c-3p and miR-302d-3p increased the number of cells arrested in the G0 and G1 phase, and decreased number of cells arrested in the S phase by flow cytometry (*p* < 0.05) (Fig. 3f). EdU assay and TUNEL assay revealed that overexpressed miR-302b-3p, miR-302c-3p or miR-302d-3p inhibited cell proliferation while promoting cell apoptosis (both *p* < 0.05) (Fig. 3g, h). These data indicated that miR-302b-3p, miR-302c-3p or miR-302d-3p could repress CSCC cell proliferation while stimulating apoptosis via downregulation of CCNO.

### miR-302b-3p, miR-302c-3p or miR-302d-3p stim ulates CSCC cell apoptosis and suppresses tumor growth by targeting CCNO in vivo

**Fig. 4:**
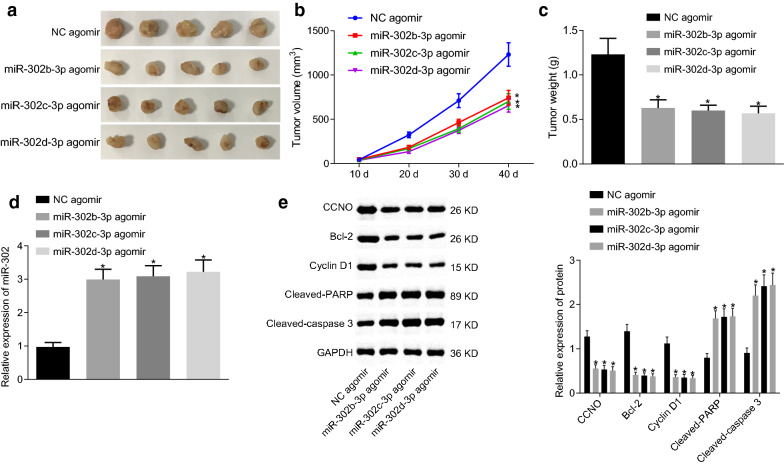
miR-302b-3p, miR-302c-3p or miR-302d-3p induces apoptosis of CSCC cells and inhibits tumor growth in vivo by targeting CCNO. **a** The tumors collected at 40 days after CasKi cell transplantation into nude mice; **b** Tumor volume in nude mice transplanted with CasKi cells overexpressing miR-302b/c/d-3p. **c** Tumor weight in nude mice transplanted with CasKi cells overexpressing miR-302b/c/d-3p; **d** miR-302b/c/d-3p expression in mice detected by RT-qPCR; **e** The protein levels of CCNO, Bcl-2, CyclinD1, cleaved PARP, and cleaved caspase 3 in mice detected by Western blot analysis, with protein bands assessed. *p < 0.05 vs. mice treated with NC agomir. The values in the figure were all measurement data, and expressed as mean ± standard deviation. One-way ANOVA was used for data comparison among multiple groups, and Tukey’s post hoc test was conducted. Repeated measures ANOVA with Bonferroni post hoc test was used for data comparison among groups at different time points. N = 05. The experiment was repeated 3 times independently

Overexpressed miR-302b-3p, miR-302c-3p or miR-302d-3p resulted in a significant decrease in size, volume and weight of subcutaneous tumors in nude mice (Fig. 4a–c). RT-qPCR showed an increase in the expression of miR-302b-3p, miR-302c-3p or miR-302d-3p in mice following the overexpression of miR-302b-3p, miR-302c-3p or miR-302d-3p (*p* < 0.05) (Fig. 4d), which was indicative of successful transfection. Western blot analysis revealed that highly-expressed miR-302b-3p, miR-302c-3p or miR-302d-3p reduced the expression of CCNO, Bcl-2, Cyclin D1, cleaved PARP and cleaved caspase 3 in subcutaneous tumors (*p* < 0.05) (Fig. 4e). The aforementioned data indicated that miR-302b-3p, miR-302c-3p or miR-302d-3p might accelerate CSCC cell apoptosis and prevent tumor growth by targeting CCNO in vivo.

#### RACK1 inhibits CCNO expression by promoting miR-302b-3p, miR-302c-3p or miR-302d-3p expression

**Fig. 5:**
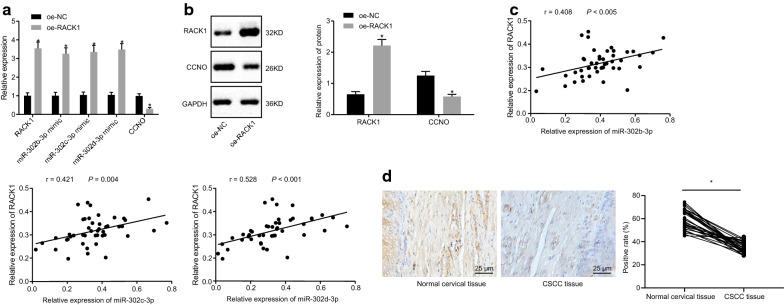
RACK1 suppresses CCNO expression through enhancement of miR-302b-3p, miR-302c -3p or miR-302d-3p expression. **a** The expression of RACK1, miR-302b/c/d-3p, and CCNO in cells detected by RT-qPCR, *p < 0.05 vs. cells treated with oe-NC; **b** Protein levels of RACK1 and CCNO examined by Western blot analysis, with protein bands assessed, *p < 0.05 vs. cells treated with oe-NC; **c** The correlation between RACK1 and miR-302b/c/d-3p in CasKi cells analyzed by Pearson’s correlation coefficient; **d** Positive expression of RACK1 protein in CSCC (N = 46) and normal cervical tissues (N = 30) detected by immunohistochemistry (×400). *p < 0.05 vs. cells treated with oe-NC. The values in the figure were measurement data, and expressed as mean ± standard deviation. Independent sample *t* test was used for data comparison between two groups. The experiment was repeated 3 times independently

RT-qPCR results showed that overexpressed RACK1 remarkably upregulated expressions of miR-302b-3p, miR-302c-3p or miR-302d-3p, while significantly downregulating CCNO expression (*p* < 0.05) (Fig. 5a). RACK1 protein level was increased and the protein level of CCNO was reduced after overexpression of RACK1 (*p* < 0.05) (Fig. 5b). Pearson’s correlation coefficient exhibited a positive correlation between RACK1 expression and the expression of miR-302b-3p, miR-302c-3p or miR-302d-3p in CasKi cells (*p* < 0.05) (Fig. 5c). In clinical settings, the positive expression of RACK1 protein was downregulated in CSCC patients from immunohistochemistry results (Fig. 5d). In summary, RACK1 inhibited CCNO expression by promoting expression of miR-302b-3p, miR-302c-3p or miR-302d-3p.

### RACK1 facilitates CSCC cell apoptosis and inhibits tumor formation in vivo in CSCC via miR-302b-3p, miR-302c-3p or miR-302d-3p-mediated CCNO inhibition

**Fig. 6:**
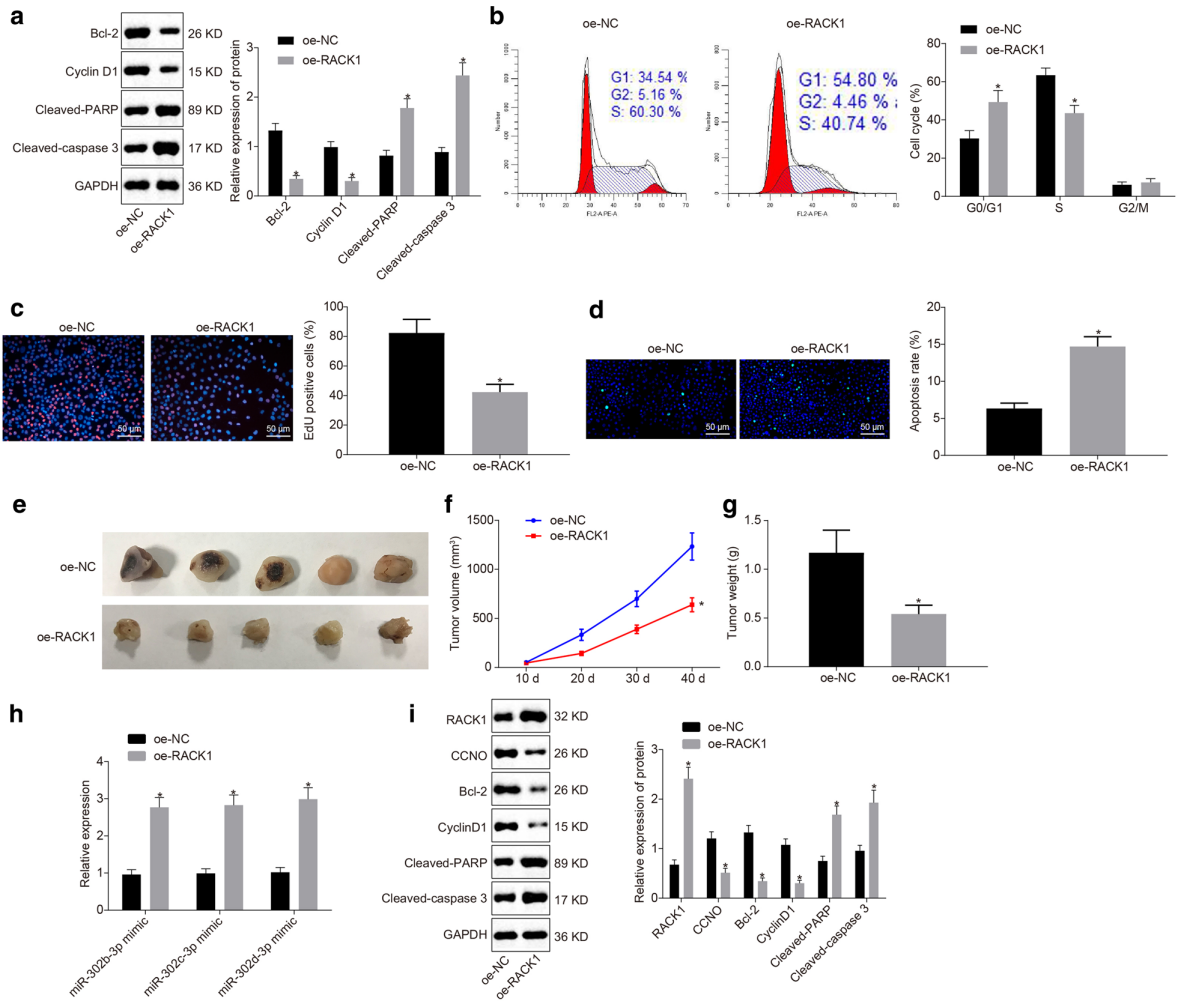
RACK1 promotes CSCC cell apoptosis and suppresses tumor growth in vivo and in vitro by regulating the miR-302b/c/d-3p-CCNO axis. **a** The protein expression of Bcl-2, Cyclin D1, cleaved PARP, and cleaved caspase 3 in CSCC cell line (CasKi) detected by Western blot analysis, with protein bands assessed; **b** The cell cycle entry of CSCC cell line (CasKi) detected by flow cytometry; **c** The cell proliferation detected by EdU assay; **d** The cell apoptosis detected by TUNEL assay; **e** The tumors collected at 40 d after CasKi cell transplantation into nude mice; **f** Tumor volume in nude mice transplanted with CasKi cells overexpressing RACK1. **g** Tumor volume in nude mice transplanted with CasKi cells overexpressing RACK1. **h** The expression of miR-302 in mouse tumors detected by RT-qPCR; I, The protein levels of RACK1, CCNO, Bcl-2, CyclinD1, cleaved PARP, and cleaved caspase 3 in mouse tumors detected by Western blot analysis, with protein bands assessed. *p < 0.05 cells or mice treated with oe-NC. The values in the figure were measurement data, and expressed as mean ± standard deviation. Independent sample *t* test was used for the data comparison between two groups. Repeated measures ANOVA with Bonferroni post hoc test was used for data comparison among groups at different time points. The experiment was repeated 3 times independently. N = 05

A series of experiments were conducted to evaluate the effects of the RACK1/miR-302b/c/d-3p-CCNO axis in CSCC cell progression as well as tumor growth. Western blot analysis results showed that overexpressed RACK1 led to a significant reduction in the expression of CCNO, Bcl-2 and Cyclin D1 and markedly elevated expression of RACK1, cleaved PARP, and cleaved caspase 3 (*p* < 0.05) (Fig. 6a).

Flow cytometry revealed that number of cells arrested in the G0 and G1 phase was increased but number of cells arrested in the S phase was reduced after overexpression of RACK1 (*p* < 0.05) (Fig. 6b). EdU assay and TUNEL assay depicted that overexpressed RACK1 induced markedly reduced cell proliferation and obviously elevated cell apoptosis (both *p* < 0.05) (Fig. 6c, d).

Furthermore, tumor xenograft in nude mice exhibited a pronounced decline in size, volume and weight of subcutaneous tumors in nude mice after overexpression of RACK1 (Fig. 6e–g). RT-qPCR results showed that expression of miR-302b-3p, miR-302c-3p or miR-302d-3p was increased significantly in response to overexpressed RACK1 (*p* < 0.05) (Fig. 6h). In addition, the protein expression of CCNO, Bcl-2, and Cyclin D1 was found to be decreased, RACK1, while that of cleaved PARP, and cleaved caspase 3 was increased after overexpression of RACK1 (*p* < 0.05) (Fig. 6I). The aforementioned findings suggested that the overexpression of RACK1 promoted CSCC cell apoptosis and suppressed tumor growth in vivo by inhibiting CCNO through regulation of miR-302b-3p, miR-302c-3p or miR-302d-3p.

## Discussion

Cervical cancer is the fourth leading cause of cancer-related deaths among females [2]. Squamous cell carcinomas, which arise from precursor squamous intraepithelial lesions, account for the majority of cervical carcinoma cases [20]. This study explored the underlying mechanism by which RACK1 is involved in CSCC and the findings demonstrated that RACK1 inhibited CCNO by promoting the expression of miR-302b/c/d-3p, thereby stimulating apoptosis of CSCC cells and delaying the progression of CSCC.

RACK1 has been previously found to be upregulated in cancer tissues obtained from 25 cervical cancer patients in comparison with the adjacent non-cancerous tissues [21]. In addition, tissue microarray in another study revealed abundant levels of RACK1 expression in squamous intraepithelial lesion and cervical cancer [22]. However, the current study demonstrated decreased RACK1 expression in cancer tissues from the collected 46 CSCC patients compared to normal cervical tissues from 30 cases. This discrepancy may be caused by the number of the recruited study subjects and the employed controls. In addition, CSCC patients presented with down-regulated miR-302b/c/d-3p and up-regulated CCNO. Downregulated miR-302-3p has been observed in cervical cancer tissues in comparison to adjacent normal tissues, and its low expression has been closely associated with node metastasis, advanced clinical stage, and poor prognosis in patients with cervical cancer [15]. Another study provided evidence that miR-302 directly targets another cyclin family member, Cyclin D1, and suppresses its expression, contributing to delayed tumorigenicity of endometrial cancer cells [23]. In the present study, CCNO was identified as the target of miR-302b/c/d-3p. In addition, elevated mRNA expression of CCNO has been reported in GC tissues and depletion of CCNO can significantly induce cancer cell apoptosis both in vitro and in vivo [18], which is partially consistent with our findings.

Our study also revealed that up-regulated RACK1 inhibited CCNO expression by promoting miR-302b/c/d-3p expression, resulting in accelerated apoptosis in CSCC cells, as evidenced by decreased Bcl-2 and Cyclin D1 expression, and increased cleaved PARP and cleaved caspase 3 expression. RACK1 is capable of upregulating the expression of a series of miRNAs, including the miR-302 cluster, and its loss promotes GC tumor invasion and metastasis through miR-302c suppression [10]. The pro-apoptotic functions of RACK1 and its ability induce apoptosis of cells, partly by inhibiting Src have been established in a previous study [24]. Up-regulated Bcl-2 contributes to the development of laryngeal squamous cell carcinoma and inhibits cell apoptosis [25]. Cyclin D1 is associated with all cell cycle and pathologic process regulation [26]. Cyclin D1 has been proven to be capable of promoting cellular proliferation [27], and has a role in the regulation of cell migration and invasion in CSCC [28]. A previous study provided evidence that cleaved PARP-1 can serve as an apoptotic marker in the proliferative regions of the spheroids [29]. The biological role of cleaved PARP-1 includes DNA repair, maintenance of genomic integrity, modulation of transcription, replication and differentia, as illustrated in a prior study [30]. There is a study confirming that cleaved caspase 3 can be induced by Tian Xian liquid, which can inhibit tumor growth and induce apoptosis [31], suggesting that cleaved caspase 3 is positively correlated with apoptosis and inhibition of tumor expansion. miR-302 is a member of the miRNA family that regulates cancer progression and invasion via a reprogramming process, which has comprehensive effects on multiple cellular pathways and events [32]. In human endometrial carcinoma cells, miR-302b/c/d-3p has been verified to stimulate the apoptotic process [33]. These findings implicated RACK1 in CSCC through the miR-302b/c/d-3p/CCNO signaling. However, further investigations are required to elucidate the potential role of CCNO in CSCC cell migration, invasion, and lymph node metastasis in order to provide an in-depth analysis of the molecular mechanism of RACK1 in CSCC.

## Conclusions

**Fig. 7:**
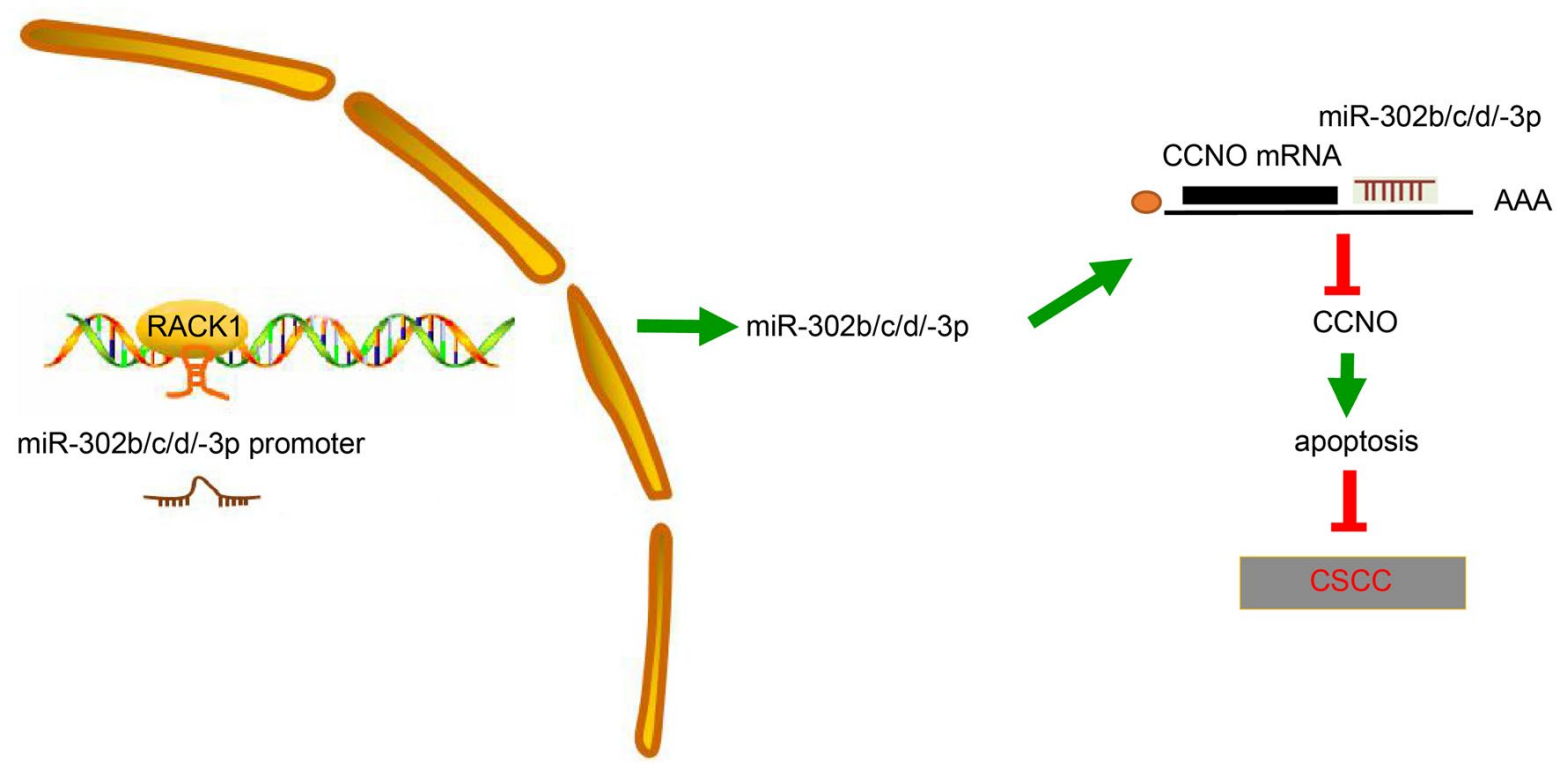
A schematic of the regulatory network of RACK1 in CSCC. RACK1 enhanced miR-302b/c/d-3p expression and then inhibited CCNO expression, thereby suppressing cell apoptosis in CSCC and consequently retarding the progression of CSCC

In conclusion, overexpression of RACK1 could potentially enhance miR-302b/c/d-3p expression and then inhibit CCNO expression, thereby inducing cell apoptosis in CSCC and ultimately preventing the progression of CSCC (Fig. 7), which provides novel therapeutic target for CSCC. However, as this study is still in the very early stages of evaluating the specific role of RACK1 in CSCC, more studies are required to further clarify its underlying mechanism and validate its applicable value in clinical practice.

## Data Availability

The data sets used and/or analyzed during the current study are available from the corresponding author on reasonable request.

## Abbreviations

CSCC: cervical squamous cell carcinoma
CCNO: Cyclin O
HPV: human papillomavirus
miRNAs: microRNAs
GC: gastric cancer
RACK1IL: receptor for activated protein kinase C
DCUN1D1: defective in cullin neddylation 1 domain containing 1
IL: interleukin
3′-UTR: 3′-untranslated region
EMEM: Eagle’s minimal essential medium
FBS: fetal bovine serum
EDTA: ethylenediaminetetraacetic acid
BNCC: BeNa Culture Collection
PARP: poly (ADP-ribose) polymerase
Bcl-2: B-cell lymphoma 2
DAB: 3,3′-diaminobenzidine
BSA: bovine serum albumin
WT: wild type
MUT: mutant type
NC: negative control
PI: propidium iodide
oe: overexpression
si: small interfering RNA
TUNEL: terminal deoxynucleotidyl transferase-mediated dUTP-biotin nick end labeling
PBS: phosphate-buffered saline
cDNA: complementary DNA
RT-qPCR: reverse transcription quantitative polymerase chain reaction
GAPDH: glyceraldehyde-3-phosphate dehydrogenase
TBST: tris-buffered saline with Tween-20
IgG: immunoglobulin G
EdU: 5-ethynyl-2′-deoxyuridine
ANOVA: analysis of variance
